# Supplementary material for: Animal Welfare Guidelines for International Development Organisations in the Global South
Source: Animals (Basel). 2024 Jul 8;14(13):2012. doi: 10.3390/ani14132012 (PMC11240469; doi:10.3390/ani14132012)
Supplement: Supplementary file 1 [file animals-14-02012-s001.zip › animals-3049363-supplementary.pdf]

APPENDIX S1: SAMPLE STANDARD OPERATING PROCEDURES (SOP)

SPECIES: …e.g. Goats………………………	NO OF ANIMALS: ……………………………	SOP CODE (if any): …………………
**PERSON(S) RESPONSIBLE:** ………………………………………………….………………………………………….…...		

CATEGORY (CT)	REQUIREMENTS (RQ)
**CT-1** (e.g. Housing)	RQ-1 (e.g. Goats will be kept in groups, except for veterinary or animal welfare reasons)
	RQ-2
	RQ-etc
**CT-2 ….**	

DATE OF FIRST ISSUE: …..…../……..../……….	DATE REVISED: …..…../…..……/…………	VERSION NO: ………………….……………
**REASON FOR REVISION:** …………………………………………………………………………………………......		
**APPROVED BY (*requires competent personnel*):** ………………………………		
